# Ω-3 fatty acids-supplementary in gestation alleviates neuroinflammation and modulates neurochemistry in rats

**DOI:** 10.1186/s12944-018-0894-2

**Published:** 2018-11-03

**Authors:** Mimi Tang, Ruili Dang, Shao Liu, Mengqi Zhang, Yi Zheng, Rui Yang, Tao Yin

**Affiliations:** 1 Department of Pharmacy, Xiangya Hospital, Central South University, Changsha, 410008 China; 2 Institute of Hospital Pharmacy, Xiangya Hospital, Central South University, Changsha, 410008 China; 3 Institute of Clinical Pharmacy, Jining First People’s Hospital, Jining Medical University, Jining, 272000 People’s Republic of China; 4 Department of Neurology, Xiangya Hospital, Central South University, Changsha, 410008 China; 5 Key Laboratory of Hunan Province for Traditional Chinese Medicine in Obstetrics and Gynecology Research, Hunan Provincial Maternal and Child Health Care Hospital, No. 53 XiangChun Road, Changsha, 410008 People’s Republic of China

**Keywords:** Ω-3 fatty acids, Neuroinflammatary factors, Purinergic type 2X7 (P2X7), NOD-like receptor pyrin domain containing 3 (NLRP3), Nuclear factor-kappaB (NF-kB), Neurotransmission

## Abstract

**Background:**

The mechanisms underlying the association between immune activation and postpartum depression remained elusive. Although Ω-3 fatty acids possess anti-inflammatory properties, there is limited evidence directly linking the modulating effects of Ω-3 fatty acids on neuroimmune and neurochemistry to the antidepressant actions.

**Methods:**

A between-groups design was used to assess the effects of reproductive status (virgin or parous) and Ω-3 fatty acids content (control and supplementary). Serum inflammatory cytokine levels (IL-1a, IL-1β, IL-2, IL-6, IL-12, TNF-a, IFN-γ) were evaluated using the Bio-Plex Luminex System. Moreover, we also measured the protein levels of Purinergic type 2X7 receptor (P2X7R), NOD-like receptor pyrin domain containing 3 (NLRP3) and Nuclear factor-kappaB (NF-κB). Lastly, we assessed the function of various neurotransmitter systems to link the inflammatory response and neurotransmitter metabolism.

**Results:**

Pro-inflammatory cyrokines, including IL-1a, IL-6, TNF-a and IFN-γ were markedly induced in the serum of parous rats, although no significantly depressive-like behavior was found. Meanwhile, NLRP3 and NF-κB were decreased in certain brain areas. Moreover, gestational stress significantly induced neurochemical disturbance, which is partly restored by Ω-3 fatty acids supplementation.

**Conclusions:**

These findings strengthen the link between inflammation, neurochemistry and postpartum depression, and further provide novel insights into the antidepressant effect of Ω-3 fatty acids.

## Background

The pathogenesis of depression has not yet been defined. Among many hypotheses, the neuroimmune theory continues to generate substantial interest. Several studies in both humans and animals have provided evidence for a link between the inflammatory process and the depressive disorders. Patients with depression were reported to exhibit an activation of the inflammatory response as shown by increased levels of proinflammatory cytokines (e.g. interleukin-1β (IL-1β), IL-6, tumor necrosis factor-a (TNF-a), interferon-γ (IFN-γ)) and altered secretion of anti-inflammatory cytokines (e.g. interleukin-4 (IL-4) and IL-10), as well as increased concentrations of acute-phase proteins in the peripheral [1–5]. Also studies involving animal models of depression have revealed alterations in the function of immune system both in the periphery and in the central nervous system (CNS). A restraint stress model in mice demonstrated a higher expression of IL-1β in the hippocampus [6]. Furthermore, in chronic mild stress (CMS) model of depression, the concentrations of IL-1β, IL-6, IL-18 and TNF-a in the brain or serum were enhanced [7–9]. It is worth emphasizing that the serum proinflammatory cytokines, including IL-1β and IL-6, were also increased in the rats with postpartum depression [10].

Omega-3 polyunsaturated fatty acids (Ω-3 PUFAs), especially eicosapentaenoic acid (EPA, C20:5n-3) and docosahexaenoic acid (DHA, C22:6n-3), are essential fatty acids that play crucial roles in balancing inflammation and neurobiological mechanisms of depression [11]. Ω-3 fatty acids and Ω-6 fatty acids consumption have opposite influence on inflammation. Arachidonic acid (AA, C20:4n-6), a Ω-6 fatty acids, increase proinflammatory cytokine production [12]. However, studies have found the association between higher intake of Ω-3 PUFAs and lower proinflammatory cytokine production [13]. Maternal storage of DHA, a biologically important LC-PUFA, was reported to be reduced during pregnancy [14]. Importantly, a recent meta-analysis has found that Ω-3 PUFAs supplementation show antidepressant effect on depression patients [15]. However, whether the reduction of Ω-3 PUFAs during pregnancy could increase the proinflammatory cytokine production, and further increase the risk of depression in parous female remains unknown.

In recent years, increased attention has been paid to the importance of cytosolic signalling pathways of inflammation. Among these, the NOD-like receptor pyrin domain containing 3 (NLRP3) inflammasome is of particular interest [16]. The NLRP3 inflammasome is activated by a wide range of divergent invading pathogens and cellular damages, and subsequently results in activation of caspase-1 by which inactive forms of IL-1β and IL-18 (i.e., pro-IL-1β and pro-IL-18) are processed to mature IL-1β and IL-18. It has been shown that LPS- and CUMS-induced depression is associated with NLRP3 inflammasome activation in brain [8, 9, 11, 17]. Patients with major depressive disorder (MDD) also reported to exhibit increased level of NLRP3 inflammasome in peripheral blood mononuclear cells [18].Given that inflammation in brain may contribute to depression-like behavior, we hypothesized that NLRP3 inflammasome may contribute to neuroinflammation in postpartum depression. Purinergic type 2X7 receptor (P2X7R) is an ionotropic receptor located predominantly on microglia and macrophages and is activated in response to cellular danger signals, such as adenosine triphosphate (ATP) [19]. Studies of peripheral immune cells demonstrate that the ATP/P2X7R-induced oligomerization of NLRP3 is the major steps of inflammatory response to danger substances [20, 21]. Stress may increase ATP, which further activates P2X7R and releases IL-1β with subsequent activation of the NLRP3 inflammasome [22].

Inflammation-induced disorder was reported to be associated with perturbations of neurotransmission, especially the imbalance between serotonin (5-HT) and kynurenine (KYN) branches of tryptophan (TRY) metabolism due to the activation of the tryptophan-degrading enzyme indoleamine 2,3-dioxygenase (IDO) [23, 24]. Moreover, increased glutamate will releases ATP, and further activates P2X7R and the subsequent inflammatory response [25]. Thus, inflammatory response may interact with neurotransmitter metabolism, and further influence the development of depression.

In the present study, we evaluated the serum levels of proinflammatory cytokines to illustrate the modulate effect of Ω-3 PUFAs on neuroimmune system. We also measured the inflammation and oxidative stress markers, P2X7R, NLRP3 and NF-kappaB (NF-κB) in the prefrontal cortex and hippocampus, which are thought to mediate the expression of pro-inflammatory factors. Finally, we analyzed neurochemical metabolites spanning amino acids, dopamine (DA), noradrenaline (NE), 5-HT and KYN metabolic pathways in the rat brain to gain further insight into the interrelationship between inflammation and neurotransmission.

## Methods

### Animals and husbandry

Adult, male and female Sprague-Dawley rats were initially housed in a temperature-controlled environment under a 12/12 h light/dark cycle with free access to food and water except prior to sucrose preference test (SPT). All efforts were made to minimize suffering. This study was approved by the Animal Care & Use Committee of Central South University. All experiments were performed in accordance with the Guide for Care and Use of Laboratory Animals (Chinese Council).

### Experimental design

A between-groups design was used to assess the effects of reproductive status (virgin or parous) and Ω-3 PUFAs content (control and supplementary). After a short acclimation period, rats were randomly assigned to groups mentioned above (*n* = 6–7). Breeding stock maintained on corresponding diets from two weeks before mating to the 3 weeks of postpartum. One male rat was housed with two female rats per cage for three days at the time of mating. To meet all current nutrient standards for rats’ pregnancy and growth [26], the Control diet in our experiment was AIN-93G (Trophic Animal Feed High-Tech Co., Ltd., China) formulated with soybean oil (70 g/kg). The Supplementary diet was identical to the Control diet except the oil formulation. The Supplementary diet was prepared with fish oil (20 g/kg) and soybean oil (50 g/kg). The fatty acids composition of the diets is shown in Table 1. After breeding treatments and behavior tests, rats were anesthetized and sacrificed.

**Table 1:** Fatty acids composition of the Experimental Diets

Fatty acids	Content in diet(area percent)	
	Control	Supplementary
C16:0	11.21	9.77
C18:0	3.59	3.41
C18:3n3	4.70	3.50
C18:1n9c	24.24	23.00
C18:2n6c	54.69	47.70
C20:5n3	ND	6.96
C22:6n3	ND	3.72
Other MUFA	1.56	1.95

Diet fatty acids composition was determined by GC/MS using Supelco 37 Standard. ND: Not detected

### Forced swinmming test (FST)

The paradigm is based on the evaluation of immobility as a measure of behavioral despair in stressful and inescapable situations. The test was performed as previously reported [27]. Two swimming sessions were conducted: a 15-min pretest on the first day followed by a 5-min test the next day. Briefly, each rat was placed in a plastic drum (45 cm height, 25 cm diameter) containing approximately 35 cm of water (24 ± 1 °C) for a 15-min pretest. After swimming, rats were dried with towels and placed back in their home cage. Twenty-four hours later, the rat was exposed to the same experimental conditions for a 5-min FST. Water was changed before each trial. Increasing immobility time is the indicator for depressive-like symptom, which was defined as floating passively and only making slight movements to keep the head above water. Each test session was videotaped and the duration of immobility was scored by two experienced observers blind to the experiment design.

### Sucrose preference test (SPT)

SPT is a measure of stressed-induced anhedonia state, a key depressive-like behavior in rats [28]. Prior to SPT, the rats were housed individually in separated cages and given free access to two bottles of sucrose solution (1%, *w*/*v*). Then after 24 h, one bottle of sucrose solution was replaced with water. On day 3, rats were deprived of water for 23 h, and then rats were given free access to two pre-weighed bottles of solution: 100 ml of sucrose solution (1%, w/v) and 100 ml of water. The side (left and right) of the bottles was randomly placed to avoid spatial bias. One hour later, the consumed volume in both bottles was recorded. The preference for sucrose was measured as a percentage of the consumed 1% sucrose solution relative to the total amount of liquid intake.

### Determination of systemic concentration of inflammatory cytokines

Serum inflammatory cytokine levels (IL-1a, IL-1β, IL-2, IL-6, IL-12, TNF-a, IFN-γ) were measured with the Bio-Plex System and Luminex xMAP technology (Bio-Rad Laboratories, Inc., USA) using a high sensitivity kit (Bio-Techne; R&D Systems, Inc., USA). The Bio-Plex 200 system (Bio-Rad Laboratories, Inc., USA) uses fluorescently dyed beads, a flow cytometer and associated optics, and a high-speed digital signal processor to detect up to 100 different types of molecules in a single well of a 96-microwell plates, requiring low sample volumes [29, 30]. The colour-coded beads are pre-coated with analyte-specific capture antibodies which bind to the cytokine of interest. Then, biotinylated detection antibodies specific to the analytes of interest are added, forming an antibody-antigen sandwich. Finally, phycoerythrin-conjugated streptavidin is added, binding to the biotinylated detection antibodies. With this technology, relevant inflammatory cytokines could be detected in a single run. Dyed beads are read on the Bio-Plex analyser. One laser classifies the bead and determines the cytokine that is being detected, and a second laser determines the magnitude of the phycoerythrin-derived signal, which is in direct proportion to the amount of molecule bound. Cytokine concentrations were derived by interpolating the measured fluorescence intensities to standard curves, and correcting by the corresponding dilution factor employed to achieve the minimum volume for analysis. Bio-Plex Manager software was employed to calculate cytokine concentrations. To avoid inter-assay variations, all samples were analyzed with the same kit on the same day.

### Western blot analysis

Protein extracts of tissues (10 μg) were mixed with gel loading buffer and separated on 12% SDS-PAGE gels. After electrophoresis, the proteins were transferred onto PVDF membranes and then blocked with 5% nonfat dry milk in Tris-buffered saline (TBS). Membranes were incubated with the following primary antibodies: anti-P2X7, anti-NALRP3, anti-NF-κB and anti-β-actin. After incubation with the primary antibodies, membranes were washed with Tris-buffered saline containing 0.05% Tween-20 (TBST), and incubated with appropriate horse radish peroxidase (HRP)-conjugated secondary antibodies. The film signal was digitally scanned and then quantified using Image J software.

### The determination of neurotransmitters

Following the method we established before [31], neurotransmitters and their metabolites were quantified using high-performance liquid chromatography coupled to tandem mass spectrometry (HPLC-MS/MS). Briefly, brain tissues were homogenized by tissue homogenizer with 1 ml of 85% ice-cold acetonitrile-water adding 10 μl of mixed internal standard solution (containing 20 μg/ml 3,4-dihydroxybenzylamine, 10 μg/ml 5-hydroxyindole-2-carboxylic acid and 100 μg/ml L-aspartic acid-13C4,15 N). After the homogenate, the mixture was centrifuged at 4 °C for 15 min at 10000 rpm. The supernatant (500 μL) was then transferred and subsequently evaporated to dryness. For derivatization, 150 μl of dansyl chloride solution (4 mg/ml in acetonitrile) and 50 μl of 0.1 M Na2CO3-NaHCO3 buffer (pH 11.0) were added to the residue and reacted at 35 °C for 30 min. After the reaction, the pH of the mixture was adjusted by adding 10 μl of 7.5% formic acid solution. After centrifugation, the supernatant was transferred to the vial for analysis. HPLC-MS/MS analysis was carried out on a Waters Acquity ultra-performance liquid chromatography system (Waters, USA) with a Micromass Quattro Premier XE tandem quadruple mass spectrometer (Waters, USA) equipped with ESI source. The chromatographic separation was achieved on Ultimate XB-C8 column, 2.1 mm × 50 mm, 3.0 μm particle size (Welch, China). The mobile phase for elution was a gradient established between solvent A (water with 20 mM ammonium acetate and 0.1% formic acid) and solvent B (acetonitrile) at a flow rate of 0.25 ml/min. The mass spectrometer was operating at the following parameters: capillary voltage, 3.00 kV; extractor voltage, 3.00 V; source temperature, 120 °C; desolvation temperature, 450 °C; desolvation gas flow, 750 L/h; cone gas flow, 50 L/h. Argon used as the collision gas was introduced into the collision cell at a flow rate of 0.16 ml/min. The electrospray ionization source was operated in the positive mode. Data acquisition was carried out by Mass Lynx 4.1 software. Neurotransmitters were quantified relative to the internal standard areas and calibrated using standard curves.

### Statistical analysis

Results from the experiment were expressed as means ± SEM and analyzed using SPSS software. Differences between groups were determined by two-way ANOVA with reproductive status (yes or no) and Ω-3 PUFAs content (control and supplementary) as main factors. When significant interaction or main effect was found for any item, post hoc analysis for multiple pairwise comparisons was performed using the Bonferroni correction. The level of significance was set at 0.05.

## Results

### Forced swimming test

**Fig. 1:**
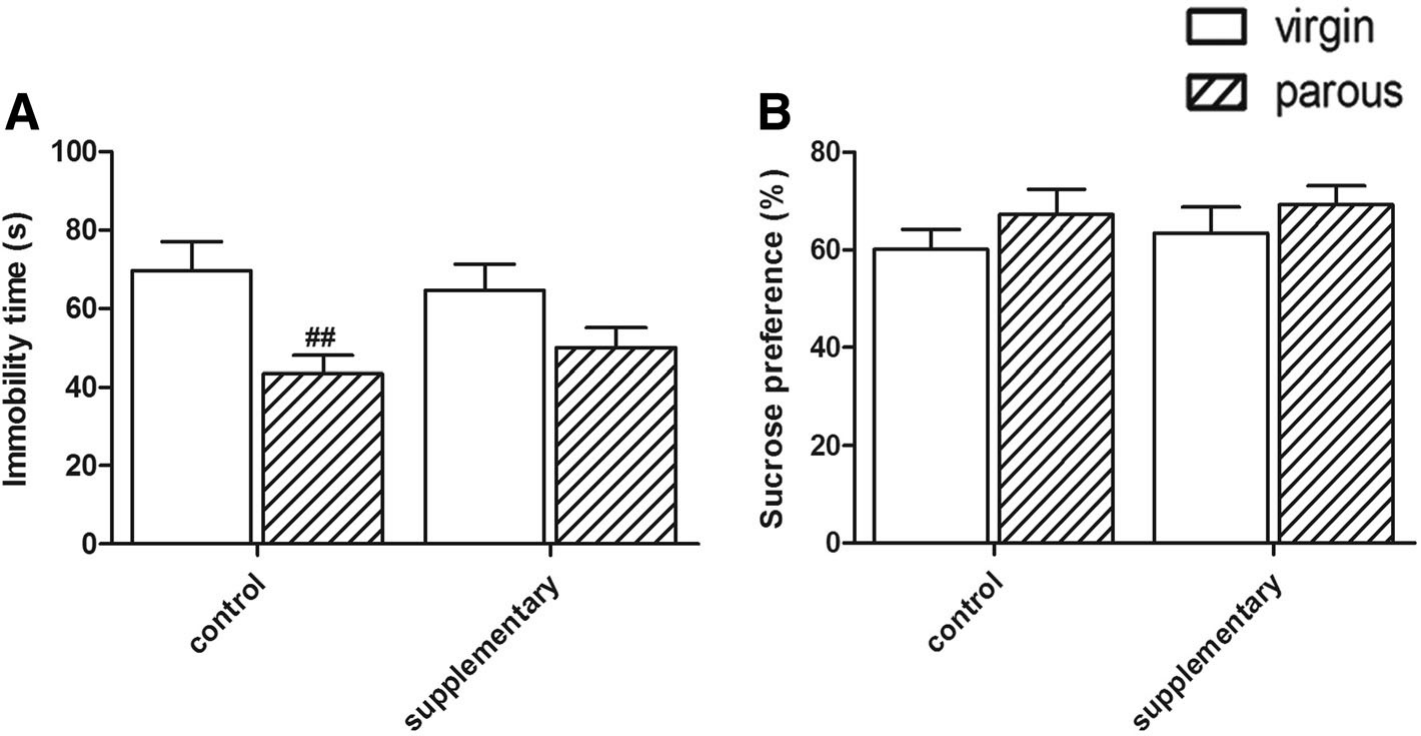
Performance in the behavior test. Immobility time in the FST (**a**); Sucrose preference in the SPT (**b**). ^#^significantly different between reproductive status conditions (Two-way ANOVA followed by LSD post hoc test, *n* = 6–7 in each group)

Two-way ANOVA on immobility time in FST indicated a significant effect of reproductive status (F(1,24) = 11.523 *p* < 0.01) (As shown in Fig. 1a). In the control groups, virgin rats had a higher immobility time than parous rats (*p* < 0.01). This effect of reproductive status was greatly attenuated in supplementary rats, resulting in no significant difference between the virgin and parous rats (*p* = 0.198).

### Sucrose preference test

As to the percentages of sucrose preference, no significantly difference was found between the virgin and parous rats in different dietary groups (Diet: F(1, 24) < 1; reproductive status: F(1, 24) < 1): CON group (virgin: 63.46 ± 5.38%; parous: 77.80 ± 3.69%), ENR group (virgin: 67.22 ± 5.23%; parous: 69.23 ± 3.93%)(Shown in Fig. 1b).

### Serum inflammatory cytokines

**Fig. 2:**
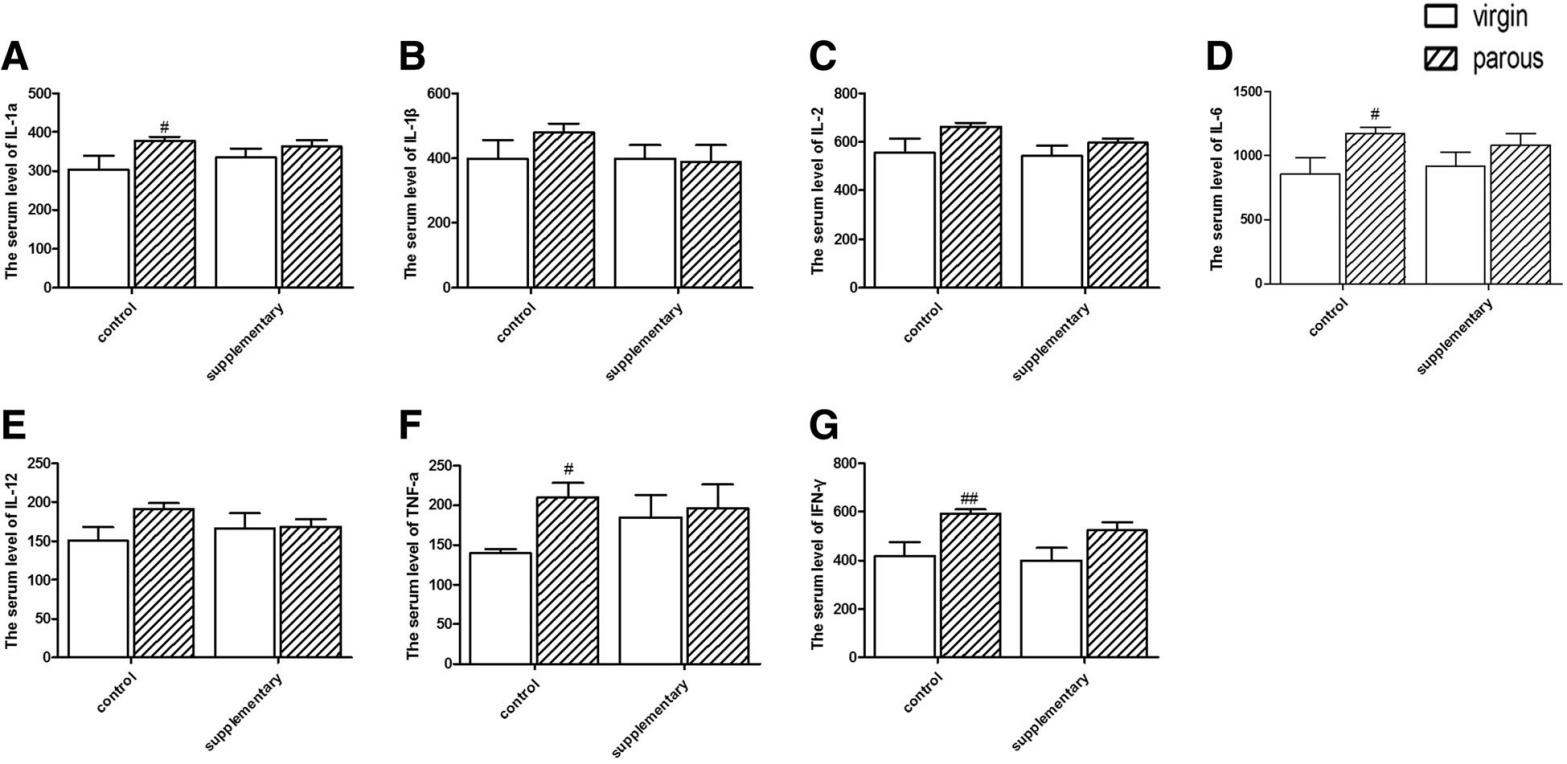
Serum levels of proinflammatory factors. Effect of reproductive status and dietary conditions on serum IL-1a (**a**); Effect of reproductive status and dietary conditions on serum IL-1β (**b**); Effect of reproductive status and dietary conditions on serum IL-2 (**c**); Effect of reproductive status and dietary conditions on serum IL-6 (**d**); Effect of reproductive status and dietary conditions on serum IL-12 (**e**); Effect of reproductive status and dietary conditions on serum TNF-a (**f**); Effect of reproductive status and dietary conditions on serum IFN-γ (**g**). ^#^significantly different between reproductive status conditions (Two-way ANOVA followed by LSD post hoc test, *n* = 6–7 in each group)

To further explore the neuroinflammatory response to parous treatment, we firstly evaluated the serum inflammatory cytokines. As shown in Fig. 2, the four pro-inflammatory cyrokines IL-1a (*p* < 0.05), IL-6 (*p* < 0.05), TNF-a (*p* < 0.05) and IFN-γ (*p* < 0.01) were markedly induced in the serum of parous rats, whereas supplementary with Ω-3 fatty acids significantly ameliorated the pregnancy-induced upregulation of these proinflammatory cyrokines.

### Protein levels of P2X7, NALRP3 and NF-κB in the prefrontal cortex

**Fig. 3:**
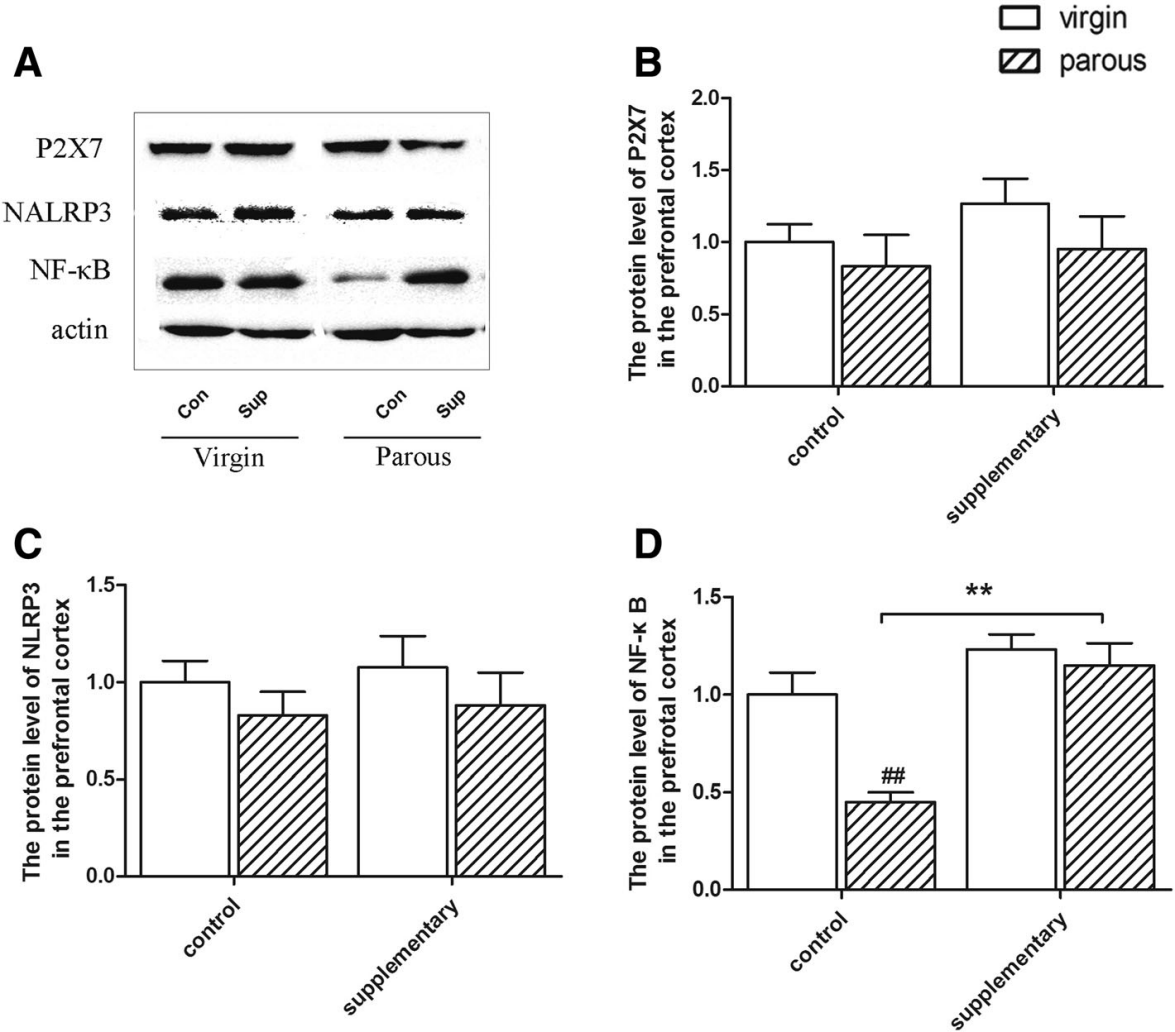
Protein levels in the prefrontal cortex. Protein expression of P2X7, NLRP3, NF-κB and β-actin in the prefrontal cortex (**a**); Effect of reproductive status and dietary conditions on protein levels of P2X7 in the prefrontal cortex (**b**); Effect of reproductive status and dietary conditions on protein levels of NLRP3 in the prefrontal cortex (**c**); Effect of reproductive status and dietary conditions on protein levels of NF-κB in the prefrontal cortex (**d**). ^#^significantly different between reproductive status conditions and *significantly different between dietary conditions (Two-way ANOVA followed by LSD post hoc test, *n* = 6–7 in each group)

The NF-κB level was influenced by diet (F(1,24) =13.429, *p* = 0.002) and reproductive status (F(1,24) = 25.324, *p* = 0.000) (Fig. 3d). In the control rats, parous rats had a lower protein level of NF-κB (*p* < 0.01). Fish oil supplementation attenuated the effect of reproductive status in control rats and significantly increased the expression of NF-κB compared to control groups (*p* < 0.01).

### Protein levels of P2X7, NALRP3 and NF-κB in the hippocampus

**Fig. 4:**
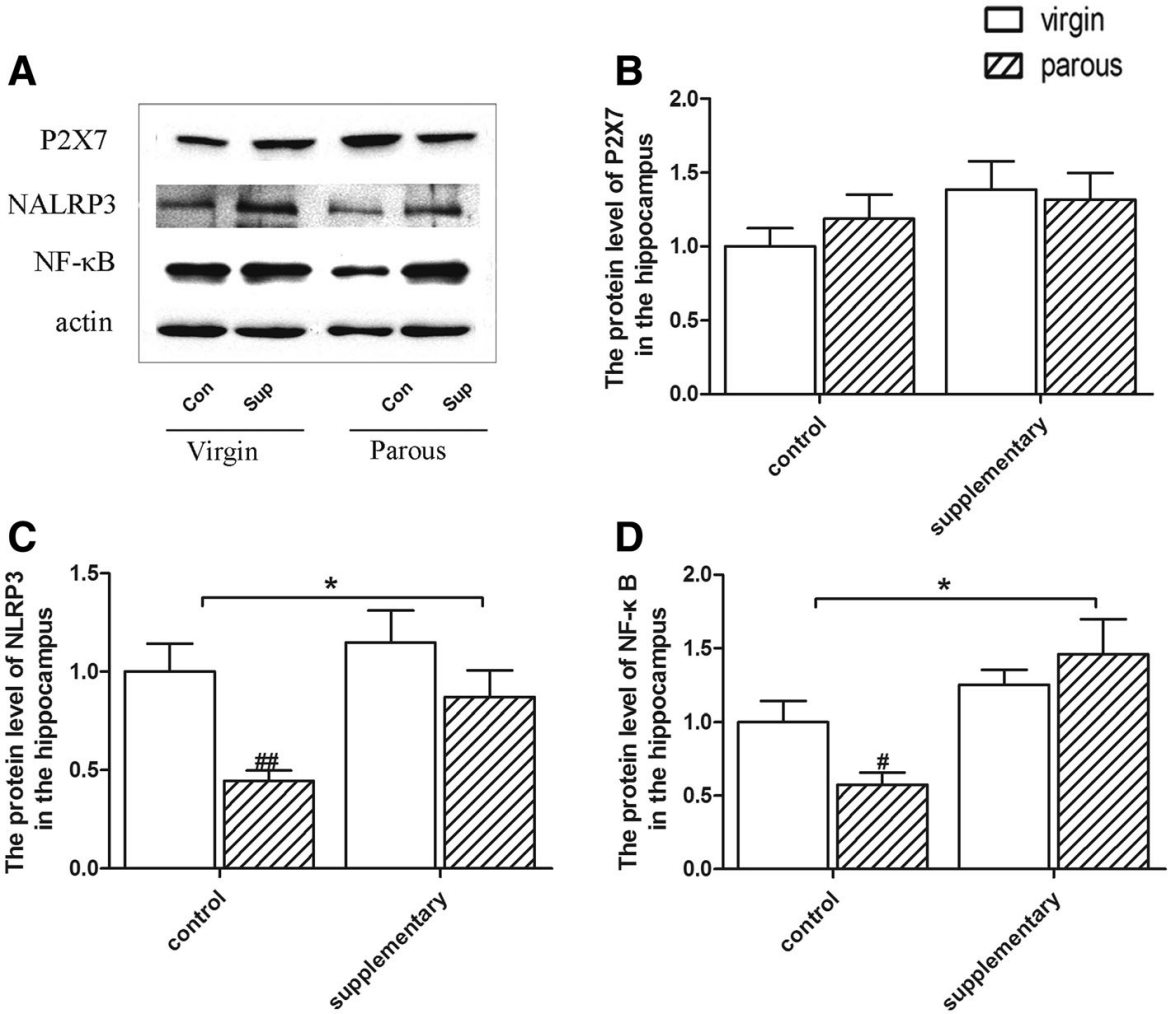
Protein levels in the hippocampus. Protein expression of P2X7, NLRP3, NF-κB and β-actin in the hippocampus (**a**); Effect of reproductive status and dietary conditions on protein levels of P2X7 in the hippocampus (**b**); Effect of reproductive status and dietary conditions on protein levels of NLRP3 in the hippocampus (**c**); Effect of reproductive status and dietary conditions on protein levels of NF-κB in the hippocampus (**d**). ^#^significantly different between reproductive status conditions and *significantly different between dietary conditions (Two-way ANOVA followed by LSD post hoc test, *n* = 6–7 in each group)

As to the protein level of P2X7, no significantly difference was found between the virgin and parous rats in different dietary groups in the hippocampus (shown in Fig. 4b). However, the protein level of NALRP3 was influenced by diet (F(1,24) = 13.095, *p* = 0.002) and reproductive status (F(1,24) = 5.305, *p* = 0.035). In the control rats, as shown in Fig. 4c, parous rats had a lower protein level of NALRP3 (*p* < 0.01). Fish oil supplementation significantly increased the expression of NALRP3 compared to control groups (*p* < 0.05). In parallel with the prefrontal cortex, the NF-κB level was influenced by diet (F(1,24) = 14.899, *p* = 0.001) (Fig. 4d) in the hippocampus. And parous rats had a lower protein level of NF-κB than virgin rats in the control groups (*p* < 0.05). Similarly, fish oil supplementation eliminated the effect of reproductive status in the expression of NF-κB.

### Brain neurochemistry

To further explore the interrelationship between parous-induced neuroinflammation and neurotransmitters, we systematically analyzed the neurochemistry both in the prefrontal cortex and in the hippocampus of rats. As shown in Tables 2 and 3, DA level were significantly decreased both in the prefrontal cortex (*p* < 0.01) and in the hippocampus (*p* < 0.05) of parous groups, while its metabolites 3,4-dihydroxyphenylacetic acid (DOPAC) and homovanillic acid (HVA) remain stable. Parous rats that exposed to control diet also exhibited decreased norepinephrine (NE, *p* < 0.01 for prefrontal cortex), without altering the metabolites vanilmandelic acid (VMA) and 4-Hydroxy-3-methoxyphenylglycol (MHPG). However, virgin rats that with daily supply of Ω-3 fatty acids exhibited decreased NE (*p* < 0.01 for prefrontal cortex) and increased VMA (*p* < 0.05 for prefrontal cortex). It was worth to mention that both parous and Ω-3 fatty acids supplementation did not affect the serotonin (5-HT) level, but parous rats that exposed to daily supplementary of Ω-3 fatty acids exhibited decreased 5-hydroxy indole acetic acid (5-HIAA, *p* < 0.01) and the 5-HT turnover (the ratio of 5-HIAA to 5-HT, *p* < 0.05). Unexpectedly, parous resulted in significant increase of γ-aminobutyric acid (GABA) status (Table 2, *p* < 0.01) and glutamine (GLN, Table 2, *p* < 0.01) in the prefrontal cortex. Conversely, we find opposite trend in the hippocampus which exhibit decrease of GABA (*p* < 0.01) and GLN (*p* < 0.01) in parous.

**Table 2:** The content of major neurotransmitters and their metabolites in the prefrontal cortex

Compound	Virgin		Parous	
	Control	Supplementary	Control	Supplementary
DA (ng/g)	5.3 ± 0.6	5.4 ± 1.2	2.2 ± 0.3^##^	3.4 ± 0.3^#^
DOPAC (ng/g)	6.8 ± 0.9	10.1 ± 1.9	10.1 ± 1.0	7.7 ± 0.5
HVA (ng/g)	1.1 ± 0.2	1.3 ± 0.3	1.3 ± 0.1	1.8 ± 0.2
NE (ng/g)	8.2 ± 1.8	3.8 ± 0.9**	3.3 ± 0.6^##^	5.9 ± 0.7
MHPG (ng/g)	0.7 ± 0.1	0.9 ± 0.1	0.9 ± 0.1	1.1 ± 0.1
VMA(ng/g)	1.2 ± 0.1	2.0 ± 0.4*	0.6 ± 0.1	0.5 ± 0.1^##^
TRY (ug/g)	9.2 ± 1.6	6.7 ± 1.4	8.2 ± 0.7	12.0 ± 1.0^##,^*
5-HT (ng/g)	153.7 ± 14.0	127.7 ± 31.3	116.0 ± 14.6	178.6 ± 26.1
5-HIAA (ng/g)	373.9 ± 45.6	355.6 ± 27.0	304.0 ± 25.8	215.3 ± 11.02^##,^*
5-HIAA/5-HT	3.0 ± 0.4	3.2 ± 0.8	2.8 ± 0.4	1.2 ± 0.2^#,^*
KYN (ng/g)	540.7 ± 99.0	429.0 ± 101.2	526.1 ± 51.4	622.2 ± 58.8
GABA (ug/g)	292.6 ± 41.5	200.3 ± 49.8	602.7 ± 82.8^##^	730.1 ± 99.2^##^
GLU (ug/g)	0.4 ± 0.06	0.3 ± 0.05	0.5 ± 0.06	0.7 ± 0.07
GLN (ug/g)	178.2 ± 25.2	132.0 ± 35.8	356.7 ± 41.5^#^	501.0 ± 64.2^##^

Data are means ± SEM (*n* = 6–7). ^#^*p* < 0.05, ^##^*p* < 0.01 compared to virgin group; *p < 0.05, **p < 0.01compared to control group

**Table 3:** The content of major neurotransmitters and their metabolites in the hippocampus

Compound	Virgin		Parous	
	Control	Supplementary	Control	Supplementary
DA (ng/g)	6.3 ± 0.8	5.0 ± 0.7	4.2 ± 0.4^#^	3.6 ± 0.5^#^
DOPAC (ng/g)	3.1 ± 0.5	2.0 ± 0.2	3.1 ± 0.7	3.5 ± 0.7
HVA (ng/g)	0.5 ± 0.1	0.4 ± 0.1	0.3 ± 0.0	0.4 ± 0.1
NE (ng/g)	3.8 ± 0.4	3.5 ± 0.5	2.7 ± 0.3	3.0 ± 0.3
MHPG (ng/g)	0.4 ± 0.0	0.3 ± 0.0	0.3 ± 0.0	0.3 ± 0.0
VMA(ng/g)	4.0 ± 0.3	3.5 ± 0.7	4.5 ± 0.4	3.5 ± 0.4
TRY (ug/g)	4.8 ± 0.3	4.0 ± 0.1	3.7 ± 0.1	4.6 ± 0.4
5-HT (ng/g)	129.6 ± 17.9	105.5 ± 14.0	108.1 ± 9.1	121.1 ± 12.3
5-HIAA (ng/g)	194.5 ± 30.3	166.8 ± 29.6	123.4 ± 9.5	71.8 ± 14.1^##^
5-HIAA/5-HT	1.8 ± 0.5	2.3 ± 0.6	0.7 ± 0.1	0.7 ± 0.2^#^
KYN (ng/g)	282.6 ± 24.5	242.4 ± 6.9	222.8 ± 8.8	239.3 ± 14.4
GABA (ug/g)	63.3 ± 5.0	49.5 ± 3.1	31.6 ± 1.5^##^	34.4 ± 1.5
GLU (ug/g)	0.1 ± 0.0	0.1 ± 0.0	0.1 ± 0.0	0.1 ± 0.0
GLN (ug/g)	48.3 ± 3.7	38.0 ± 3.7	29.2 ± 1.3^##^	32.5 ± 1.1

Data are means ± SEM (*n* = 6–7). ^#^*p* < 0.05, ^##^*p* < 0.01 compared to virgin group; *p < 0.05, **p < 0.01compared to control group

## Discussion

In the present study, we established a model of normal pregnancy to evaluate the depressive-like behavior, the dysregulated neuroimmune system and neurotransmitter system in parous rats. In particular, we demonstrated that Ω-3 fatty acids could attenuate the proinflammatory cytokines and modulate neurotransmitter system. Besides, our study also showed that the turbulence of neuroimmune system manifested in parous rats might have some relation to the immunological defects, which can featured by reduced protein level of NF-κB.

The FST is an extensively validated predictor of antidepressant efficacy, and has been used as a putative model simulating depressive behavior in rodents [32]. Increasing immobility time is the indicator for depressive-like symptom. In the present study, parous dams exhibited less immobility time than virgin females, which was different with previous study [33]. We speculate that the different terminal point of observation can partly explain the divergence. SPT is also a key behavioral indicator to depressive-like behavior. Unfortunately, no significantly difference was found in the SPT between the virgin and parous rats in different dietary groups. Postpartum rats were reported to drink almost 93% sucrose at PND 25, and then reduced to 65% at PND180 [34], which suggest that it may needs longer time to form the state of anhedonia.

Numerous studies have suggested that major depression is accompanied by immune dysregulation. Proinflammatory cytokines have been shown to induce stress-reactive neuroendocrine and central neurotransmitter changes in depression. As previously reported, the proinflammatory cytokines TNF-α and IL-6 in depressed subjects were significantly higher than control subjects. However, the levels of some markers of inflammation, such as C-reactive protein, TNF-a, IL-1, IL-2 and IL-8 differ between studies [35]. However, the serum proinflammatory cytokines, including IL-1β and IL-6, were increased in the rats with postpartum depression [10]. In the present study, four pro-inflammatory cyrokines including IL-1a, IL-6, TNF-a and IFN-γ were markedly induced in the serum of parous rats, which reflected the activation of neuroimmune system under gestational stress. Elevated cytokines may play an important role in depression for following reasons: modulate hippocampal neurogenesis [36]; induce the IDO enzyme [37] and impact the hypothalamic-pituitary-adrenal (HPA) axis [35, 38]. Our data showed that Ω-3 fatty acids alleviated the alterations of proinflammatory cytokines induced by pregnancy, lending more weight to hypothesis that anti-depressive action of Ω-3 fatty acids is through their potent immunomodulating effects.

The physiological role of NF-κB is best delineated in the immune system. Either the under- or over-activation of NF-κB has the capacity to result in dysregulated inflammation [39]. Knockout mice for the NF-κB show predominantly immunological defects [40]. Moreover, the absence of p50, the consist member of NF-κB, leads to enhanced NK cell proliferation and production of IFN-γ [41]. In the present study, we find the reduced expression of NF-κB both in the prefrontal cortex and in the hippocampus of parous rats, along with the decreased serum IFN-γ. We speculated that the reduction of NF-κB reflected immune system disorders in parous rats and Ω-3 fatty acids supplementary successfully reversed gestational stress-induced alterations in rats. Similarly with NF-κB, NLRP3 also induced by a wide range of divergent stress and has been reported to couple with NF-κB inflammatory signaling to mediate transcription and function of proinflammatory cytokines [11]. Limiting the activation of NLRP3 could inhibit the inflammation induced by divergent invading pathogens and cellular damages [42, 43]. However, we didn’t find any studies that regarding the association of the potential effect of NLRP3 knockdown with immune system. Further studies are needed to figure out the possible reason why NLRP3 decreased in the hippocampus of parous rats. As mention before, ATP/P2X7R-induced oligomerization of NLRP3 is the major steps of inflammatory response. P2X7R/NLRP3 inflammasome axis has also been demonstrated to link cytokine, psychological stress and depression [11]. However, we didn’t find activated P2X7R/NLRP3 axis in parous rats. Instead, we find decreased NF-κB and NLRP3 in certain brain areas. Other pathways may be involed in the activation of proinflammatory cytokines in parous rats.

The alterations of neurotransmission in the key brain areas play a pivotal role in the progression of neuropsychiatric disease, and the beneficial effects of Ω-3 fatty acids in these brain-related disorders was, at least partially, via its modulating effect on neurotransmissions [44]. Our data showed that parous-induced alterations of multiple neurotransmitters systems, including DA, NE, 5-HT and glutamate systems. DA plays a key role in governing motivation and reward processing. In line with previous report, the brain content of DA in parous rats was also decreased in our study [45]. Along with the reduction of DA, the precursor of NE, we also find decreased NE in the prefrontal cortex of parous female rats. In addition, the content of 5-HT in the prefrontal cortex of parous rats was decreased, but it was not significant. Tryptophane (TRY), the precursor of 5-HT also can be metabolized to KYN through indoleamine 2,3-dioxygenase (IDO) which is activated by inflammatory cytokines resulting in accelerated conversion of TRY to KYN and reduced bioavailability of TRY for 5-HT production [46]. In the present study, the content of KYN was increased in parous rats that exposed to Ω-3 fatty acids diet, without change the 5-HT and KYN levels, which imply the relatively stable of TRY metabolism. It is worth to mention that the content of 5-HIAA and the ratio of 5-HIAA/5-HT (5-HT turnover) were significantly decreased in the prefrontal cortex and the hippocampus of parous rats with Ω-3 fatty acids supplementary diet. Previous work has shown increased 5-HT turnover in the prefrontal cortex of gestational stress, and antidepressant fluoxetine treatment could normalize the alterations and reduce the 5-HIAA/5-HT ratios [47]. Additionally, the use of antidepressant and improvement of depression was reported to associate with the reduction of 5-HT turnover [48]. Thus, it is possible that Ω-3 fatty acids supplementary may exhibit protective effect via its modulating effect in serotonin system. Abnormalities in GLU and GABA signal transmission also have been postulated to play a role in depression. GABA is the major inhibitory neurotransmitter in the central nervous system, which is produced in the CNS via decarboxylation of GLU. Within the CNS, the majority of GLU is produced from GLN via the enzyme glutaminase. Reduced GABA levels have been observed in the cortex of depressed patients [49] or hippocampal of rats exposed to chronic mild stress [50]. In the present study, we find decreased GABA in the hippocampus rather than in the prefrontal cortex. However, the concentration of GLU remains stable. Also, Ω-3 fatty acids supplementary failed to reverse these alterations.

There are some limitations in the present study. First, we didn’t determine the cytokines in pre-frontal cortex and hippocampus. Thus, we fail to complete the correlation analysis of cytokines and NF-κB or NLRP3 levels. Second, we didn’t add the analysis of phosphorylated portion of NF-κB to our paper. Third, we didn’t establish a typical model of postpartum depression. Additional research is needed to figure out the changes of neuro-immune system and potential regulatory mechanisms.

## Conclusion

Our data showed that instead of inducing depressive-like behaviors, gestational stress significantly induced the serum levels of proinflammatory cytokines and perturbations of neurotransmitter system, which may ultimately contribute to the pathology of inflammation-induced depression. An important finding in the present study is that Ω-3 fatty acids attenuated the gestational stress-induced neuroinflammation. Concomitant with reduced neuroinflammation, Ω-3 fatty acids also involved in modulating the dysregulation of neurotransmission system. These results provide more insight into the link between the neuro-immune modulating features of Ω-3 fatty acids and their potential antidepressant actions.

## Abbreviations

5-HIAA: 5-hydroxy indole acetic acid
5-HT: Serotonin
AA: Arachidonic acid
CMS: Chronic mild stress
CNS: Central nervous system
DA: Dopamine
DHA: Docosahexaenoic acid
DOPAC: 3,4-dihydroxyphenylacetic acid
EPA: Eicosapentaenoic acid
FST: Forced swinmming test
GABA: γ-aminobutyric acid
GLN: Glutamine
GLU: Glutamate
HPA: Hypothalamic-pituitary-adrenal
HPLC-MS/MS: High-performance liquid chromatography coupled to tandem mass spectrometry
HRP: Horse radish peroxidase
HVA: Homovanillic acid
IDO: Indoleamine 2,3-dioxygenase
IFN-γ: interferon-γ
IL: Interleukin
KYN: Kynurenine
MDD: Major depressive disorder
MHPG: 4-Hydroxy-3-methoxyphenylglycol
NE: Noradrenaline
NF-κB: Nuclear factor-kappaB
NLRP3: NOD-like receptor pyrin domain containing 3
P2X7R: Purinergic type 2X7 receptor
PUFAs: Polyunsaturated fatty acids
SPT: Sucrose preference test
TNF-a: Tumor necrosis factor-a
TRY: Tryptophan
VMA: Vanilmandelic acid
